# Optimizing single-port thoracoscopic anatomical sublobar resection of the right upper lobe with computed tomography three-dimensional (3D) reconstruction technology: a retrospective cohort study

**DOI:** 10.3389/fsurg.2025.1702827

**Published:** 2025-12-10

**Authors:** Yang Tian, Baisheng Xie, Nannan Song, Jun Wang

**Affiliations:** Department of Cardiothoracic Surgery, Hangzhou Hospital of Traditional Chinese Medicine, Hangzhou, China

**Keywords:** anatomical sublobar resection, ground glass nodule, computed tomography three-dimensional (3D) reconstruction technology, thoracoscope, sublobes of the lung

## Abstract

**Objective:**

To investigate the viability of assisting with single-port thoracoscopic anatomical sublobar resection of the right upper lobe of the lung using computed tomography three-dimensional (3D) reconstruction technology.

**Methods:**

From January 2021 to January 2024, 152 cases of single-port thoracoscopic anatomical sublobar excision of the right upper lobe of the lung were recorded in our hospital's Department of Thoracic Surgery. Depending on whether the 3D reconstruction technique was employed, the cases were split into two groups: one for 3D and one for 2D. The final analysis included 65 pairs (130 cases) of patients after applying 1:1 propensity score matching to compensate for baseline confounding factors. Surgical time, intraoperative blood loss, margin distance, postoperative complications, and other perioperative indicators were all statistically examined and reported.

**Results:**

The 3D group had significantly shorter surgical times than the 2D group (111.0 ± 19.4 vs. 142.7 ± 11.4, *P* < 0.001). The margin distance was significantly longer in the 3D group than in the 2D group (2.4 ± 0.4 vs. 2.1 ± 0.6, *P* = 0.017); Postoperative complications were significantly fewer in the 3D group than in the 2D group [7 [10.7%] vs. 16 [24.6%], *P* = 0.039]; The chest tube retention time was significantly shorter in the 3D group than in the 2D group (4.9 ± 1.4 vs. 5.6 ± 1.2, *P* = 0.005); The hospital stay was significantly shorter in the 3D group than in the 2D group (6.4 ± 1.5 vs. 7.1 ± 1.2, *P* = 0.005). Subgroup analysis showed that in the subgroup with the primary surgeon performing fewer than 140 procedures, the 3D group had significantly shorter surgical time and shorter hospital stay than the 2D group. In the subgroup with ≥140 surgeries performed by the primary surgeon, the 3D group had a significantly shorter surgical time, shorter hospital stay, and shorter hospital stay than the 2D group.

**Conclusion:**

Single-port thoracoscopic anatomical sublobar excision of the right upper lobe, utilizing 3D reconstruction technology, can significantly reduce surgical time, increase the surgical margin threshold, minimize hospital stays and chest tube retention times, and decrease perioperative surgical complications.

## 1 Introduction

The diagnosis of early-stage lung cancer, especially ground-glass opacities (GGOs), has increased dramatically with the widespread use of low-dose spiral computed tomography screening and high-resolution computed tomography (1). While anatomical lobectomy has become the standard surgical procedure for early-stage lung cancer, increasing evidence suggests that for stage I non-small cell lung cancer (NSCLC), anatomical segmentectomy offers comparable short- and long-term outcomes to anatomical lobectomy while preserving more lung function and improving patients' quality of life (2, 3). In contrast to lobectomy, anatomical sublobar resection is more anatomically complex and has a higher rate of variability, necessitating more exacting surgical methods to guarantee sufficient safety margins and segmental plane confirmation. It is challenging to meet the requirements of such precise surgery because traditional computed tomography imaging is limited in its ability to define anatomical boundaries and determine the relative positions of pulmonary arteries, veins, trachea, and nodules. Consequently, 3D reconstruction technology for computed tomography has been developed (4).

Each of the three segments (S1, S2, and S3) that make up the right upper lobe has two subsegments (a and b). The right upper lobe's anatomy is relatively simple in comparison to other lobes, which makes it a prime candidate for anatomical sublobar resection. The right upper lobe also has a large number of small inter-segmental veins that drain into the central veins, as well as a variety of artery variations. Each segment and subsegment of the right upper lobe's bronchi, arteries, and veins can be seen in three dimensions using 3D reconstruction technology. This is particularly useful for displaying the tiny branches of the inter-segmental veins (V1a, V1b, V2a, V2b, etc.), which are often missed in conventional two-dimensional imaging. By enabling multi-angle rotation and observation, 3D models help prevent unintentional injuries during surgery. 3D navigation helps surgeons to anticipate the type of variation in advance for common arterial variations in the right upper lobe (like A1 + A2 common trunk or A3 independent origin), which lowers surgical risk and difficulty while improving perioperative safety.

We gathered data from 152 cases of single-port thoracoscopic anatomical sublobar resection of the right upper lobe performed in our department between January 2021 and January 2024, splitting the cases into a 3D group and a two-dimensional (2D) group. To eliminate any confounding variables that might affect the results, we included all baseline variables that could potentially affect the outcomes, such as gender, age, smoking history, cardiovascular disease, chronic obstructive pulmonary disease, tumor size, and tumor location. 65 matched pairs were successfully obtained after 1:1 propensity score matching (PSM), yielding a total sample size of 130 cases, then the study assessed the feasibility and applicability of anatomical sublobar resection assisted by 3D reconstruction technology.

## 2 Materials and methods

### 2.1 General data

A retrospective analysis was conducted on the clinical and pathological data of 130 patients who underwent single-port thoracoscopic anatomical sublobar resection of the right upper lobe between January 2021 and January 2024 in our department. Among them, 36 were male (27.7%) and 94 were female (72.3%); Ages ranged from 24 to 58 years, with a median age of 48 years; 65 cases were in the 3D surgery group and 65 cases in the 2D surgery group; Postoperative pathology confirmed 4 cases (3.1%) of benign tumors, 5 cases (3.8%) of atypical hyperplasia, 58 cases (44.6%) of *in situ* adenocarcinoma, 49 cases (37.7%) of minimally invasive adenocarcinoma, 9 cases (6.9%) of invasive adenocarcinoma, 1 case (0.8%) of mucinous adenocarcinoma, 1 case of squamous cell carcinoma (0.8%), and 3 cases of metastatic tumors (2.3%).

### 2.2 Preoperative localization procedure and surgical indications

The two groups of patients adopted different preoperative localization strategies. Patients in the 2D group underwent CT-guided percutaneous puncture with medical glue localization under local anesthesia before surgery. Briefly, under local anesthesia, a coaxial puncture needle was advanced to the vicinity of the target nodule under CT guidance. Upon confirmation of the optimal needle tip position, approximately 0.1–0.3 mL of medical-grade tissue glue (n-butyl cyanoacrylate) was injected as the needle was withdrawn, forming a visible glue cast along the needle tract and adjacent to the nodule. This cast served as the tactile and visual marker during subsequent video-assisted thoracoscopic surgery (VATS). Immediately after the procedure, a follow-up CT scan was performed to confirm the success of localization and to screen for any immediate complications, such as pneumothorax or hemorrhage. All localization procedures were performed by an experienced interventional radiologist. This minimally invasive procedure aimed to provide a reference for intraoperative navigation through the medical glue marker. Patients in the 3D group did not require this additional step, and their intraoperative localization relied entirely on real—time navigation based on preoperative three—dimensional reconstruction images.

The following criteria are met by our department's anatomical sublobar resection indications, according to the National Comprehensive Cancer Network (NCCN) guidelines: (1) Nodule diameter ≤2 cm and computed tomography shows ≥50% ground-glass appearance (GGO); (2) The patient is in poor general condition and cannot tolerate lobectomy; and (3) Isolated pulmonary metastasis, in a deep location that makes wedge resection challenging.

### 2.3 Computed tomography 3D reconstruction technique

The use of 3D reconstruction was based on preoperative imaging availability and surgeon preference during the early phase of implementation. Over time, it became routinely used for all eligible patients undergoing sublobar resection. All patients in the 3D group received preoperative 3D reconstruction using Mimics software, while the 2D group underwent surgery based on conventional CT images.

**Figure 1:**
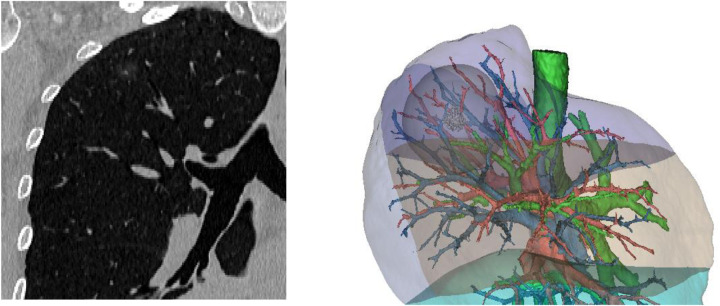
3D reconstruction of the right upper lobe and 2 cm safety margin reconstruction.

All the 3D Group patients underwent 1.5 mm chest contrast-enhanced computed tomography scanning using multi-slice spiral computed tomography before surgery. The image sequences were then transferred to a computer in Digital Imaging and Communications in Medicine (DICOM) format. Using Mimics version 21 software, the nodule, pulmonary artery, veins, bronchial branches, and inter-segmental planes were reconstructed and distinguished by different colors, with a 2 cm margin beyond the nodule simulated as the surgical safety margin (see Figure 1). After reconstruction, the surgeon and attending physician assess the results to clarify the anatomical relationships between arteries, veins, nodules, and segmental planes, aiding in surgical treatment Figure 1.

### 2.4 Surgical method

All enrolled patients were treated by the same surgical team. Double-lumen endotracheal intubation was performed, and patients were positioned in the left lateral decubitus position. A 3 cm surgical incision was made at the fourth intercostal space along the right anterior axillary line as a single operative port. An elastic cutting protection sleeve was placed at the incision site. A 10 mm 30° thoracoscope was used, and additional incisions or conversion to open surgery were selected based on the difficulty of the surgical procedure. During surgery, the target bronchus and arteries/veins were dissected and ligated using 3D lung reconstruction. The “expansion-collapse method” was used to determine the inter-segmental plane. Finally, the target lung tissue was completely dissected using a disposable cutting stapler, and lymph node sampling/cleaning was performed at the 2nd, 4th, 7th, and 10th groups. After confirming no pulmonary air leakage, one chest tube was left in place, and the surgery was concluded.

### 2.5 Statistical methods

Statistical analysis was performed using SPSS 27.0 software. A 1:1 propensity score matching was used to balance baseline differences between groups. Quantitative data were expressed as mean ± standard deviation and compared between groups using *t*-tests. Categorical variables were expressed as case numbers (proportion), *n* (%), and compared using chi-square tests. (Continuity correction or Fisher's exact test was applied as needed.) Multiple linear regression analysis was used to identify factors influencing intraoperative bleeding and surgical time. *P* < 0.05 was considered statistically significant.

## 3 Results

### 3.1 Comparison of clinical and pathological characteristics between the 2D group and the 3D group

There were no statistically significant differences between the two groups in terms of gender, age, comorbidities, tumor size, tumor pathological type, or site of surgical resection (*P* > 0.05) Table 1.

**Table 1:** Comparison of clinical characteristics between the 2D group and the 3D group.

Clinical Characteristics	2D Group (*n* = 65)	3D Group (*n* = 65)	*P*
Age (years), mean ± SD	47.8 ± 6.6	47.8 ± 6.0	0.945
Gender, *n* (%)			0.695
Male	17 (26.2%)	19 (29.2%)	
Female	48 (73.8%)	46 (70.8%)	
Comorbidities, *n* (%)			0.611
None	34 (52.3%)	40 (61.5%)	
Hypertension	11 (16.9%)	11 (16.9%)	
Diabetes	5 (7.7%)	7 (10.8%)	
Chronic obstructive pulmonary disease (COPD)	2 (3.1%)	1 (0.8%)	
Heart disease	5 (7.7%)	2 (3.1%)	
History of malignant tumors	8 (12.3%)	4 (6.2%)	
Tumor size (cm), mean ± SD	1.0 ± 0.3	1.0 ± 0.4	0936
Tumor pathological type, *n* (%)			0.960
Benign tumor	2 (3.1%)	2 (3.1%)	
Atypical hyperplasia	2 (3.1%)	3 (4.6%)	
In situ adenocarcinoma	31 (47.7%)	27 (41.5%)	
Microinvasive adenocarcinoma	23 (35.4%)	26 (40.0%)	
Invasive adenocarcinoma	4 (6.2%)	5 (7.7%)	
Mucinous adenocarcinoma	1 (1.5%)	0 (0.0%)	
Squamous cell carcinoma	0 (0.0%)	1 (1.5%)	
Metastatic tumor	2 (3.1%)	1 (1.5%)	
Site of surgical resection, *n* (%)			0.846
S1a	5 (7.7%)	2 (3.1%)	
S1b	0 (0.0%)	1 (1.5%)	
S1	15 (23.1%)	12 (18.5%)	
S2a	2 (3.1%)	1 (1.5%)	
S2b	2 (3.1%)	4 (6.2%)	
S2	24 (36.9%)	21 (32.3%)	
S3b	0 (0.0%)	1 (1.5%)	
S3	12 (18.5%)	13 (20.0%)	
S1 + 2a	1 (1.5%)	1 (1.5%)	
S1 + 2	2 (3.1%)	5 (7.7%)	
S2 + 1a	2 (3.1%)	3 (4.6%)	
S2b + 3a	0 (0.0%)	1 (1.5%)	

### 3.2 Comparison of perioperative indicators between the 2D and 3D groups

The 3D group had significantly shorter surgical times than the 2D group (111.0 ± 19.4 vs. 142.7 ± 11.4, *P* < 0.001); the 3D group had significantly longer margin distances than the 2D group (2.4 ± 0.4 vs. 2.1 ± 0.6, *P* = 0.017); postoperative complications (Clavien-Dindo grade ≥II) were significantly fewer in the 3D group than in the 2D group [7 (10.7%) vs. 16 (24.6%), *P* = 0.039]; the duration of chest tube placement was significantly shorter in the 3D group than in the 2D group (4.9 ± 1.4 vs. 5.6 ± 1.2, *P* = 0.005); the 3D group had a significantly shorter hospital stay than the 2D group (6.4 ± 1.5 vs. 7.1 ± 1.2, *P* = 0.005); there were no significant differences between the two groups in terms of intraoperative blood loss, conversion to open thoracotomy rate, or localization accuracy Table 2.

**Table 2:** Comparison of perioperative indicators between the 2D group and the 3D group.

Postoperative comparison indicators	2D Group (*n* = 65)	3D Group (*n* = 65)	*P*
Surgical time (min), mean ± SD	142.7 ± 11.4	111.0 ± 19.4	<0.001
Intraoperative blood loss (mL), mean ± SD	106.1 ± 26.9	105.2 ± 44.0	0.884
Conversion to open thoracotomy, *n* (%)	4 (6.2%)	1 (1.5%)	0.365
Marginal distance (cm), mean ± SD	2.1 ± 0.6	2.4 ± 0.4	0.017
Localization accuracy rate, *n* (%)	65 (100%)	65 (100%)	–
Postoperative complications, *n* (%)			0.039
No complications	49 (75.4%)	58 (89.3%)	
Total postoperative complications	16 (24.6%)	7 (10.7%)	
Pulmonary infection	5 (7.7%)	3 (4.6%)	
Cardiac disease	2 (3.1%)	1 (1.5%)	
Pulmonary air leak (>5 days)	7 (10.8%)	2 (3.1%)	
Hemoptysis	1 (1.5%)	0 (0.0%)	
Postoperative bleeding requiring reoperation	1 (1.5%)	1 (1.5%)	
Chest tube drainage duration (d), mean ± SD	5.6 ± 1.2	4.9 ± 1.4	0.005
Hospital stay (days), mean ± SD	7.1 ± 1.2	6.4 ± 1.5	0.005

### 3.3 Multivariate analysis of surgical time

A multivariate linear regression equation was constructed using age, gender, smoking, COPD, 3D reconstruction technology, and surgical procedure (lung segment or sub-segment). The results showed that 3D reconstruction technique (*β* = −0.737, *P* < 0.001) and the surgical procedure (*β* = 0.182, *P* = 0.004) are related to the duration of the surgery. The 3D reconstruction technique can significantly shorten the operation time, and subsegmentectomy requires significantly longer operation time than pulmonary segmentectomy Table 3.

**Table 3:** Multivariate analysis of surgical time.

Variables	*B*	Beta	*P*
Age	−0.249	−0.070	0.265
Gender	−0.516	−0.010	0.872
Smoking	0.832	0.016	0.801
COPD	−1.878	−0.013	0.842
3D reconstruction technology	−32.951	−0.737	<0.001
Surgical procedure	9.657	0.182	0.004

### 3.4 Subgroup analysis of perioperative key outcomes between the 2D and 3D groups

To further explore the impact of surgeon differences on outcomes, we combined the learning curve of single-port video-assisted thoracic surgery (5) and conducted a subgroup analysis based on the number of surgeries performed by the primary surgeon (<140 vs. ≥140). After propensity score matching, the subgroup with <140 surgeries included 25 pairs of samples in the statistical analysis, and the subgroup with ≥140 surgeries included 34 pairs of samples in the statistical analysis. We compared the effects of 3D reconstruction technology on key outcomes (blood loss, surgery time, postoperative complications, and extubation time) within each subgroup.

The analysis showed: In the subgroup with <140 surgeries performed by the primary surgeon, the 3D group had significantly shorter surgery times than the 2D group (113.2 ± 68.8 vs. 145.6 ± 11.2, *P* < 0.001), and the 3D group had significantly shorter hospital stays than the 2D group (6.3 ± 1.4 vs. 7.2 ± 1.4, *P* = 0.031). In the subgroup with ≥140 primary surgeries, the 3D group had significantly shorter surgery times than the 2D group (109.2 ± 17.5 vs. 141.6 ± 11.1, *P* < 0.001), the 3D group had a significantly shorter chest tube retention time than the 2D group (4.7 ± 1.0 vs. 5.6 ± 1.0, *P* < 0.001), and the 3D group had a significantly shorter hospital stay than the 2D group (6.3 ± 1.2 vs. 7.2 ± 1.2, *P* = 0.003) Tables 4, 5.

**Table 4:** Comparison of key outcomes between the 3D reconstruction technique and the 2D group in the subgroup with less than 140 primary surgeries.

Variables	2D Group (*n* = 25)	3D Group (*n* = 25)	*P*
Intraoperative blood loss (mL), mean ± SD	112.2 ± 40.6	113.2 ± 68.8	0.946
Surgery time (min), mean ± SD	145.6 ± 11.2	113.5 ± 22.4	<0.001
Postoperative complications, *n* (%)	4 (16.0%)	0 (0%)	0.110
Chest drain retention time (days), mean ± SD	5.5 ± 1.5	4.8 ± 1.3	0.096
Hospital stay (days), mean ± SD	7.2 ± 1.4	6.3 ± 1.4	0.031

**Table 5:** Comparison of key outcomes between the 2D and 3D groups in the subgroup with ≥140 surgeries performed by the primary surgeon.

Variables	2D Group (*n* = 34)	3D Group (*n* = 34)	*P*
Intraoperative blood loss (mL), mean ± SD	101.7 ± 10.7	99.7 ± 14.1	0.506
Surgery time (min), mean ± SD	141.6 ± 11.1	109.2 ± 17.5	<0.001
Postoperative complications, *n* (%)	12 (35.3%)	6 (17.6%)	0.099
Chest drain retention time (days), mean ± SD	5.6 ± 1.0	4.7 ± 1.0	<0.001
Hospital stay (days), mean ± SD	7.2 ± 1.2	6.3 ± 1.2	0.003

## 4 Discussion

As of right now, we've made significant progress in lung cancer early screening, detection, diagnosis, and treatment (6). For stage I non-small cell lung cancer, anatomical sublobar resection in conjunction with mediastinal lymph node dissection/sampling has emerged as the main surgical strategy (7–9). Anatomical sublobar resection minimizes the safe and complete resection of the lesion, maximizes the preservation of lung function, reduces perioperative complications, and improves long-term biochemical quality. The thoracic surgeons who perform anatomical sublobar resection surgery must, however, possess a thorough understanding of sublobar anatomy, the relationships between the trachea, arteries, and veins of lung segments and subsegments, as well as the intricate and frequently variable anatomical structures. Additionally, traditional computed tomography images are unable to provide the surgeons with a 3D understanding of pulmonary anatomical relationships, which may increase surgical risks (10).

**Figure 2:**
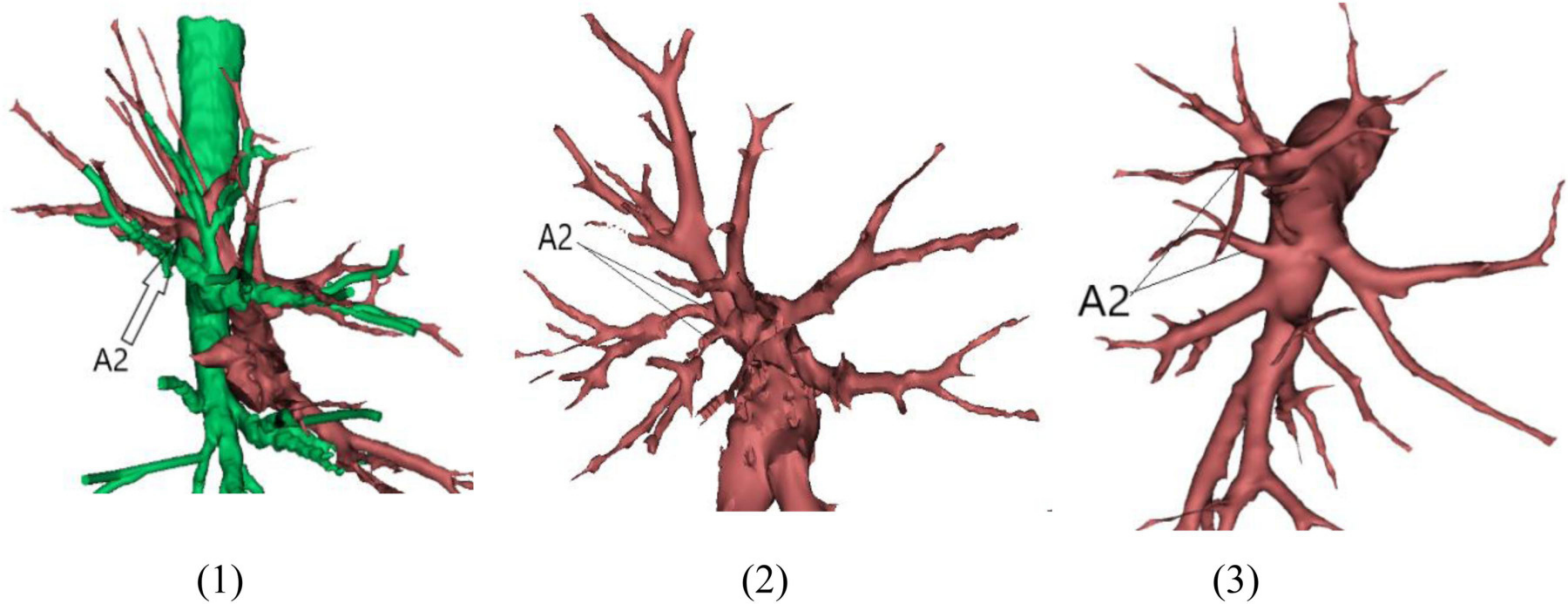
A1 + A2 common trunk.

With the growing popularity of anatomical sublobar resection in recent years, computed tomography 3D reconstruction technology has emerged as one of the main instruments for the procedure due to its distinct benefits (11). Each of the three segments (S1, S2, and S3) that make up the right upper lobe has two subsegments (a, b). The right upper lobe is a prime candidate for anatomical sublobar resection because of its comparatively simple anatomy in comparison to other lung lobes. The central vein receives venous drainage from the entire S2 segment, the majority of the S3 segment, and occasionally a small portion of the S1 segment. This is the most characteristic aspect of venous drainage in the right upper lobe. Although numerous, the inter-segmental veins that empty into the central vein have a tiny diameter. Arterial variations are also common, especially in the posterior segment, where variations like an independent origin of A3 or a common trunk of A1 + A2 may occasionally be present in addition to the most common posterior ascending artery. During surgery, the anatomical relationships between the trachea and arteries/veins, as well as the inter-segmental planes of lung segments and subsegments, can be precisely displayed using computed tomography 3D reconstruction technology. This can be compared with the actual anatomy to further clarify anatomical localization and achieve precise resection Figure 2.

Numerous studies have demonstrated that 3D reconstruction technology offers advantages over conventional surgery, including shorter operative times and reduced perioperative complications. Liu's reported that the 3D group undergoing thoracoscopic anatomic sublobar resection had significantly reduced intraoperative blood loss, shorter operative times, and a lower incidence of postoperative hemoptysis compared to the 2D group (12); She's study showed that the 3D group had significantly shorter surgical time and postoperative chest tube retention time, as well as significantly reduced intraoperative blood loss and postoperative drainage volume, compared to the 2D group, and also had significantly lower rates of postoperative hemoptysis and pneumothorax (13). The results of our study showed that the surgical time in the 3D group was significantly shorter than that in the 2D group (111.0 ± 19.4 vs. 142.7 ± 11.4, *P* < 0.001), and the incidence of postoperative complications (Clavien-Dindo grade ≥II) was significantly lower than that in the 2D group [7 (10.7%) vs. 16 (24.6%), *P* = 0.039], and the duration of chest tube placement was significantly shorter in the 3D group than in the 2D group (4.9 ± 1.4 vs. 5.6 ± 1.2, *P* = 0.005). Multivariate analysis showed that 3D reconstruction technology (*β* = −0.737, *P* < 0.001) is correlated with the time of the surgery. The 3D reconstruction technology can significantly shorten the surgery duration. Subsegmentectomy requires significantly longer surgery time than pulmonary segmentectomy. Our study is consistent with the aforementioned studies (12, 13) in terms of surgical time, complications, and chest tube retention time, which is attributed to the advantages of 3D reconstruction technology in providing 3D and precise preoperative planning. Through 3D visualization models, surgeons can more clearly identify anatomical variations in pulmonary segment vessels and bronchi, reducing the time required during surgery to identify sublobar anatomy by expanding the anatomical range of surrounding tissues. This enables more precise separation and cutting during surgery, minimizing damage to normal lung parenchyma, thereby confirming the optimized tissue protection effects of this technology; It is worth noting that the aforementioned studies (12, 13) all indicated that the 3D group had significantly less intraoperative bleeding than the 2D group, while in our study, there was no difference in intraoperative bleeding between the 2D and 3D groups, Multivariate analysis also did not show a correlation between 3D reconstruction technology and intraoperative bleeding, This might be due to the following reasons: Firstly, all the participating surgeons in this study have already surpassed the learning curve of thoracoscopic segmental lung resection. They also possess excellent anatomical identification skills and hemostasis techniques under traditional 2D imaging, thus controlling the bleeding volume in the 2D group at an extremely low level, narrowing the window for the difference between the groups. Secondly, the intraoperative bleeding volume may no longer be a sufficiently sensitive indicator in today's mature minimally invasive surgical system; the advantages of 3D technology are more likely to be reflected in improving surgical efficiency (as confirmed by this study, such as the shortened operation time) and handling complex and variable structures with greater confidence, rather than further reducing the already negligible bleeding. Therefore, our results indirectly confirm that even without relying on 3D reconstruction, lung segment resection can be safely and effectively completed through traditional 2D imaging in high-level medical centers. However, the surgical process optimization and time efficiency brought by 3D technology remain clear and clinically valuable. In addition to surgeon differences, surgical proficiency may be related to the surgical site. This study only included samples from right upper lobe anatomical sublobar resection, which, combined with the unique vascular anatomy of the right upper lobe—where interlobar venous branches are thin, prone to injury during surgery, and have high vascular variability—and the relatively limited surgical maneuvering space, these factors may have offset the advantage of 3D reconstruction technology in reducing bleeding, thereby narrowing the difference in bleeding volume between the two groups.

Regarding the safety and feasibility of computerized tomography 3D reconstruction-assisted video-assisted thoracoscopic surgery (VATS), Wu YJ enrolled 123 patients who underwent computed tomography 3D reconstruction-assisted VATS anatomical sublobar resection or lobectomy, The average duration of chest tube placement was 3.5 ± 1.6 days, the postoperative hospital stay was 6.8 ± 1.8 days, and postoperative complications included 1 case of pneumonia and 4 cases of prolonged air leakage lasting >5 days. There were no cases of intraoperative massive bleeding, postoperative intensive care unit admission, or 30-day mortality (14). Additionally, a study by Wu WB included 47 patients who underwent thoracoscopic combined segmentectomy guided by computed tomography 3D reconstruction technology, The average surgical time was 190.8 ± 54.9 min, with an average intraoperative blood loss of 42.7 ± 23.0 mL, and a postoperative thoracic drainage duration of 3.0 ± 1.8 days. And postoperative hospital stay was 5.3 ± 2.4 days (15). In our study, the 3D group had an average surgical time of 111.0 ± 19.4 min, intraoperative blood loss of 105.2 ± 44.0 mL, and a postoperative complication rate (Clavien-Dindo grade ≥II) of 10.7%, the duration of chest tube placement was 4.9 ± 1.4 days, and the length of hospital stay was 6.4 ± 1.5 days. In terms of surgical time, the study by Wu WB (15) reported a longer average surgical time than our study (190.8 ± 54.9 min vs. 111.0 ± 19.4 min), which may be attributed to differences in the types of surgeries included and the complexity of the procedures. Wu WB's study focused on combined segmentectomy (CSS), which involves managing multiple segmental planes, resulting in a broader anatomical scope and more complex procedures; in contrast, our study primarily included single-segment or segmentectomy procedures, which have a relatively limited surgical scope. Additionally, Wu WB's study was published earlier, and the longer surgical time may reflect that the center was in the early stages of applying 3D reconstruction technology. The intraoperative blood loss in our study (105.2 ± 44.0 mL) was higher than that in Wu WB's study (42.7 ± 23.0 mL). Potential influencing factors include: Wu WB's study had a smaller sample size (*n* = 47), differences in blood loss measurement methods, and our study strictly limited the procedures to right upper lobe surgeries (due to the unique vascular anatomy of the right upper lobe). Despite these differences, the three studies showed high consistency in key safety indicators, with the incidence of severe postoperative complications (Clavien-Dindo ≥Grade II) remaining at a low level (10.7% in our study vs. 4.1% in Wu YJ's study), no perioperative deaths reported, and postoperative hospital stays concentrated within the 5–7-day range, suggesting that 3D reconstruction technology has a stable rehabilitative effect across different medical centers.

Considering potential differences between authors and the impact of varying surgical experience levels on study outcomes, we conducted subgroup analyses based on the number of primary surgeries performed (<140 vs. ≥140) to compare the effects of 3D reconstruction techniques on key outcomes (blood loss, surgical time, postoperative complications, and extubation time) within each subgroup. Results showed: In the subgroup with primary surgeon caseload <140 cases, the 3D group had significantly shorter surgical time than the 2D group (*P* < 0.001), and the 3D group had significantly shorter hospital stay than the 2D group (*P* = 0.031). In the subgroup with ≥140 primary surgeries, the 3D group had significantly shorter surgical times than the 2D group (*P* < 0.001), significantly shorter chest tube drainage times than the 2D group (*P* < 0.001), and significantly shorter hospital stays than the 2D group (*P* = 0.003). It is evident that even in the presence of surgeon variability, 3D reconstruction technology can improve surgical and perioperative outcomes to varying degrees. A single-center design may not fully reflect the software dependency, surgical experience levels, or imaging tool differences among surgeons at other institutions. However, the perioperative outcomes in our study, including blood loss, surgical time, postoperative complications, hospital stay, and chest tube retention time, remain within the same range as those reported in numerous previously published studies (10, 12–16) remain within a similar range despite differences in surgeons and imaging tools. The consistency of these indicators across different settings suggests that our study results may have broader applicability, though this requires validation in a wider range of contexts. The right upper lobe has a relatively straightforward anatomical structure compared to other lobes. This is precisely why we selected it as the initial model to evaluate the feasibility and added value of 3D reconstruction technology in anatomical sublobar resection. By controlling for anatomical complexity, we were better able to isolate the effect of 3D guidance on surgical outcomes. We plan to extend this research to other lobes in future multicenter studies to validate the generalizability of our findings.

Currently, there are numerous methods for lung nodule localization, including computed tomography-guided wire localization, computed tomography-guided medical glue localization, and methylene blue staining localization. These methods are suitable for simpler wedge resections, but they are inaccurate for nodules distant from the visceral pleura and cannot precisely display the anatomical location of the nodule (17). Computed tomography 3D reconstruction technology not only avoids complications such as hemothorax, pneumothorax, and air embolism caused by needle localization but also accurately locates the segment or even subsegment where the nodule is located, ensuring sufficient surgical margins while preserving inter-segmental veins (2). Studies indicate that, under the premise of negative surgical margins, adequate surgical margins are of significant importance in preventing margin recurrence. Typically, a 20 mm margin in an expanded lung and a 15 mm margin in a collapsed lung are considered safe and feasible (18). A prospective multicenter study by Sawabata N showed that a margin distance greater than the tumor diameter is considered the optimal choice to avoid margin recurrence (19). In our study, the 2D group underwent computed tomography-guided medical glue localization preoperatively, while the 3D group was localized based on preoperative reconstruction. The results showed that the localization accuracy rate was 100% in both groups. The margin distance in the 3D group was longer than that in the 2D group (2.4 ± 0.4 vs. 2.1 ± 0.6, *P* = 0.017). The margin distance in the 3D group was 0.3 cm longer than that in the 2D group. Although this difference was statistically significant, the absolute difference was small. This may be because the margin distance was measured on ex vivo specimens, and lung tissue atrophy and collapse can shorten the margin distance. The margin distance in the 2D group was 2.1 ± 0.6 cm, which already met the 2 cm safe margin distance. while the 3D group further increased the margin distance threshold. This was primarily due to the 3D reconstruction of segmental planes and nodules with a 2 cm safe margin using computed tomography 3D reconstruction technology, enabling surgeons to grasp the relative positions of the safe margin and segmental planes in 3D space. Preoperative planning of the resection range based on margin distance helped ensure adequate margin distance. While we acknowledge that minor differences in a single indicator may not be sufficient to support broad conclusions, the consistent results across multiple studies in key safety indicators, combined with the discussion section, collectively support the application value of 3D reconstruction technology in certain medical scenarios. This is particularly relevant for anatomically complex cases or surgeons with limited surgical experience.

## 5 Conclusion

Computed tomography 3D reconstruction technology-assisted single-port thoracoscopic anatomical sublobar resection of the right upper lobe significantly reduces surgical time, increases the surgical margin threshold, shortens chest tube retention time and hospital stay, and reduces perioperative surgical complications. Combined with relatively simple anatomical characteristics, the right upper lobe is an ideal lung segment for performing computerized tomography 3D reconstruction-assisted anatomical sublobar resection, with the potential for widespread implementation in primary care hospitals. Although known confounding factors were controlled for using PSM and subgroup analysis, unmeasured variables (such as surgeon experience, preferences, and instrument differences) may still influence the results. Future large-scale, multicenter studies are needed to confirm these findings.

## Data Availability

The original contributions presented in the study are included in the article/Supplementary Material, further inquiries can be directed to the corresponding author.
